# Development and Application of a Chemical Ionization Focusing Integrated Ionization Source TOFMS for Online Detection of OVOCs in the Atmosphere

**DOI:** 10.3390/molecules28186600

**Published:** 2023-09-13

**Authors:** Ruidong Liu, Yingzhe Guo, Mei Li, Jing Li, Dong Yang, Keyong Hou

**Affiliations:** Environment Research Institute, Shandong University, Qingdao 266237, China

**Keywords:** chemical ionization source, segmented quadrupole, oxygenated volatile organic compounds, chemical ionization, photoionization

## Abstract

Single photon ionization (SPI) based on vacuum ultraviolet (VUV) lamps has been extensively investigated and applied due to its clean mass spectra as a soft ionization method. However, the photon energy of 10.6 eV and photon flux of 10^11^ photons s^−1^ of a commercial VUV lamp limits its range of ionizable analytes as well as its sensitivity. This work designs a chemical ionization focusing integrated (CIFI) ionization source time-of-flight mass spectrometry (TOFMS) based on a VUV lamp for the detection of volatile organic compounds (VOCs) and oxygenated volatile organic compounds (OVOCs). The photoelectrons obtained from the VUV lamp via the photoelectric effect ionized the oxygen and water in the air to obtain the reagent ions. The ion–molecule-reaction region (IMR) is constituted by a segmented quadrupole that radially focuses the ions using a radio-frequency electric field. This significantly enhances the yield and transport efficiency of the product ions leading to a great improvement in sensitivity. As a result, a 44-fold and 1154-fold increase in the signal response for benzene and pentanal were achieved, respectively. To verify the reliability of the ionization source, the linear correspondence and repeatability of benzene and pentanal were investigated. Satisfactory dynamic linearity was obtained in the mixing ratio range of 5–50 ppbv, and the relative standard deviation (RSD) of inter-day reached 3.91% and 6.26%, respectively. Finally, the CIFI−TOFMS was applied to the determination of OVOCs, and the LOD of 12 types of OVOCs reached the pptv level, indicating that the ionization source has the potential for accurate and sensitive online monitoring of atmospheric OVOCs.

## 1. Introduction

The ionization source of mass spectrometry determines the properties, quantity, and degree of the fragmentation of ions. Electron ionization (EI) [1] is a traditional ionization source for gas chromatography–mass spectrometry (GC-MS) [2] that is widely used in volatile organic compounds (VOCs) analysis. However, its high-energy electrons of 70 eV result in the generation of numerous ion fragments, overlapping peaks in mass spectrometry, and difficulties in qualitative and quantitative analysis. Therefore, it is replaced by various soft ionization sources such as chemical ionization (CI) [3] and photoionization (PI) [4]. Among these soft ionization sources, vacuum ultraviolet single photon ionization (SPI) has advantages such as fewer fragment ions, no sample pretreatment requirements, and minimal influence on material polarity, which makes it suitable for online analysis in the atmosphere.

The principle of SPI is the threshold ionization technology [5]. When the photon energy emitted by the light source is higher than the ionization energy (IE) of the analyte molecule, the analyte molecule can lose an electron and be directly ionized. The ionization process [6] that occurs is as follows: $M+hv\to M^{+*}+e^{-}$. Currently, vacuum ultraviolet (VUV) krypton lamps are commonly used in SPI to generate photons with energies of 10.0 eV and 10.6 eV when the breakdown threshold of the filling gas is exceeded [7]. However, the photon energy of 10.6 eV, the upper limit of a commercialized VUV krypton lamp, limits the range of its ionizable analytes. To address these issues, many new photoionization technologies have been developed in recent years, including the ones as follows: (1) designing new high-power, high-intensity discharge VUV lamps to improve the luminous flux of VUV lamps [8,9,10]; (2) improving the ionization efficiency or utilization rate of ions [11,12]; and (3) developing reagent-assisted chemical ionization sources based on VUV lamps to increase the range of ionizable analytes and improve instrument sensitivity [13,14]. These above-mentioned VUV lamp-based ionization sources have been widely used for VOC detection [15].

Oxygenated volatile organic compounds (OVOCs) [16] are mainly composed of aldehydes and ketones (carbonyls), alcohols (alcohols), ethers (ethers), low-molecular organic acids (acid), organic esters (ester), as well as extremely reactive aldehydes, enkephalins, and compounds. The mixing ratio level of OVOCs varies greatly in the ambient atmosphere, and the typical species (e.g., formaldehyde) can be as high as dozens of ppbv, and high-carbon aldehydes and ketones such as glutaraldehyde and hexanal are less than 0.1 ppbv. OVOCs are important in tropospheric chemistry as they can influence the oxidative potential of the lower troposphere, are precursors of the secondary organic aerosols, and have negative effects on human health. The wide range of mixing ratios of OVOCs in the atmosphere, their high reactivity, and the extremely short lifetimes of many species place very stringent requirements on their analytical measurement methods: to realize the analysis of OVOCs at the trace level, the temporal resolution of the measurement must be high, and the sampling and analytical processes must avoid the degradation of the target analytes or their transformation by chemical reactions. To date, no measurement method has been recognized as being able to accurately measure OVOCs in air. The development and application of a simple, fast, and accurate measurement method is an important prerequisite for the study of OVOCs.

This paper designs a chemical ionization focusing integrated (CIFI) ionization source based on a VUV lamp and radially focused ions inside the ionization source. The oxygen and the water in the air were used as the source of reagent ions in chemical ionization, and a segmented quadrupole constituted the IMR and radially focused the ions using a radio-frequency electric field. The radio-frequency (RF) voltage, ionization source pressure, and the reliability of the ionization source were comprehensively studied. Finally, the quantitative performances of a homemade time-of-flight mass spectrometer equipped with a CIFI ionization source (CIFI−TOFMS) and its application in the online monitoring of atmospheric OVOCs were investigated.

## 2. Materials and Methods

### 2.1. Chemicals

The standard gas of 1 ppmv benzene in N_2_, 1 ppmv pentanal in N_2_, and 1 ppmv OVOCs (acetaldehyde, acrolein, acetone, propionaldehyde, crotonaldehyde, methacrolein, 2-butanone, butanal, pentanal, hexanal, benzaldehyde, and m-tolualdehyde) in N_2_ were all purchased from the Dalian Special Gas Company (Dalian, China). The standard gas mixture of benzene, toluene, and p-toluene diluted with N_2_ (99.9993% purity) at calibrated mixing ratios of 1 ppmv was purchased from the Dehai Gas Company (Qingdao, China). Samples with different mixing ratios were prepared by diluting the standard gas with purified dry air. The purified dry air without NO, NOx, O_3_, SO_2_, CO, and hydrocarbons was generated using a zero-gas generator (Thermo Scientific 55i Thermo Scientific, Waltham, MA, USA).

### 2.2. Instrument and Analytical Process

**Figure 1:**
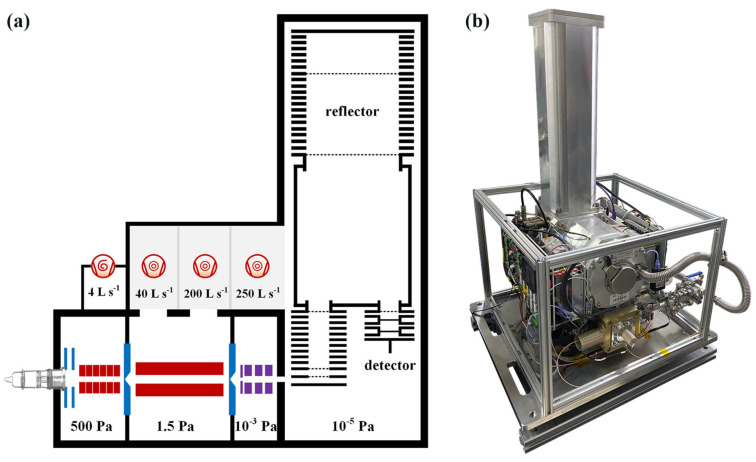
(**a**) Schematic diagram of the main components of CIFI−TOFMS; (**b**) photo of the CIFI−TOFMS.

The experimental work of the CIFI ionization source was carried out on the homemade time-of-flight mass spectrometer (TOFMS) in our laboratory. As shown in Figure 1a, the CIFI−TOFMS mainly consists of a CIFI ionization source, a quadrupole, an ion lens, a time-of-flight (TOF) mass analyzer, and a vacuum system. The vacuum system of the CIFI−TOFMS contained four differentially pumped stages. A dry pump (Leybold SCROLLVAC 15 plus) and a multi-inlet molecular pump (Leybold TURBOVAC TW 250/200/40; flow rate: 40 L s^−1^, 200 L s^−1^, and 250 L s^−1^) maintained four differentially pumped stages of the instrument, with pressure of 550, 1.5, 10^−3^, and 10^−5^ Pa, respectively.

#### 2.2.1. Design of the Chemical Ionization Focusing Integrated Ionization Source

**Figure 2:**
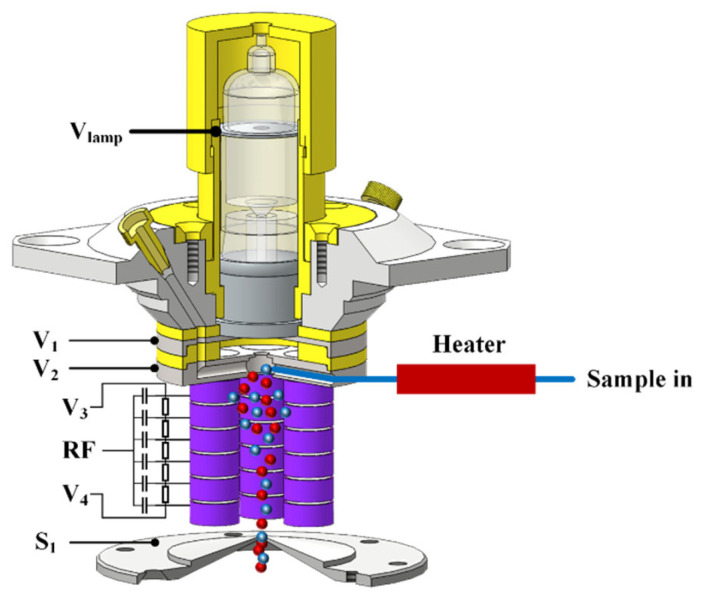
The diagram of chemical ionization focusing integrated ionization source.

Figure 2 shows a 3D cross-sectional view of the CIFI ionization source; it is mainly composed of a VUV krypton lamp, a repulsion electrode, an extraction electrode, a sample inlet, a segmented quadrupole, and a differential vacuum orifice. The vacuum ultraviolet krypton lamp was sealed with the repulsion electrode via an O-ring, and the two were placed coaxially. The center of the repulsion electrode had a 10 mm hole with the same diameter as the light window of the VUV krypton lamp. The center aperture of the extraction electrode was 3 mm, which was conducive to the collision of photoelectrons sputtered from the metal surface of the extraction electrode by the vacuum ultraviolet krypton lamp with oxygen and water in the air under the acceleration of the electric field to generate reagent ions. The sample molecules and reagent ions then entered the segmented quadrupole under the effect of pressure and electric field, which produced chemical ionization between the reagent ions and the sample molecules. At the same time, with the continuous collision between ions and molecules, the energy of ions gradually decreased and converged on the central axis before passing through the differential vacuum orifice.

The segmented quadrupole was composed of 4 rods, which were arranged in a circular array along the axis of the repulsive electrode. Each rod was composed of 6 electrode rings (5 mm inner diameter; 9 mm outer diameter; and 4 mm thickness) and was fixed on the polyetheretherketone (PEEK) insulation pole. Each electrode ring was isolated by a PEEK insulation ring (5 mm inner diameter; 9 mm outer diameter; 0.5 mm thickness). The segmented quadrupole electrode rings of each rod applied a DC voltage via a series of 10 MΩ resistances, forming a DC electric field along the axis direction. At the same time, capacitors with the same capacitance value (100 nF) were connected to apply RF voltage, forming a quadrupole field. The voltage difference between the front end of the rod and the back end of the rod was 5 V. In addition, all metal electrodes in the CIFI ionization source were treated with gold plating to prevent pollution during long-term use of the CIFI ionization source and ensure the stability of the CIFI−TOFMS.

The CIFI ionization source was the first stage vacuum, and gas was sampled from the atmosphere via a PEEK capillary with a 1 m long and 0.5 mm inner diameter. The capillary was equipped with a heating device, and the sample flow rate was about 0.2 SLM. The CIFI ionization source was pumped by a 4 L s^−1^ dry scroll pump, with a pressure adjustable between 200 and 800 Pa, and throttled down to achieve a sampling pressure of 550 Pa.

#### 2.2.2. Ion Transmission System

The ion beams generated in the CIFI ionization source entered the RF-only quadrupole through the first differential vacuum orifice (1 mm in the center). The quadrupole was made using stainless steel rods (131 mm in length), and the radii of the rods were 6.0 mm for the quadrupole. The pressure of the quadrupole chamber was maintained at around 1.5 Pa, which mainly played a role in cooling and focusing ions, thereby improving the ion transport efficiency and enhancing the sensitivity of the instrument. RF generators for the segmented quadrupole and the RF-only quadrupole were constructed in-house. A special resonance coil was a major part of the RF generator, which restricted the frequency adjustment and could only be scanned based on the capacitance value of the quadrupole to obtain a resonant frequency value. The generator output voltage was adjustable from 0 to 800 V (peak-to-peak).

The cooled ion beams entered the ion lens through the second differential vacuum orifice (2 mm in the center). The ion lens was composed of four stainless steel electrode rings to focus and drive the ion beam into the repelling region of the TOF mass spectrometer. The pressure of the ion lens chamber was maintained at below 10^−3^ Pa, which ensured that the ion beams could not collide with the background gas frequently during transmission.

#### 2.2.3. TOF Mass Analyzer

Ions with stray trajectories were prevented from entering the TOF mass analyzer by a 2 mm slit. The TOF mass analyzer was a vertically introduced reflective structure with a resolving power of over 1800 (*m*/*z* = 78), consisting of a modulator–accelerator, a drift region, a reflector, and a microchannel plate (MCP) detector. The working cycle of the modulator–accelerator consisted of two states: (1) Ions were collected in the modulator and extracted from the modulator via the accelerator into the drift region by applying short voltage pulses. (2) During the next state of ions collection, the new ions filled the modulator, while the ions from the previous collection stage passed through the drift zone, entered the reflector, were reflected, and hit the MCP detector. The drift region remained at an electric potential of −3800 V. It could protect ions from other external magnetic or electric fields during flight. The homogeneous electrical fields in the reflector were created in a series of metal rings, and the generation of the homogeneous electric field in the accelerator was similar to its structure. There was a first grid at the export of the drift region and a second grid in the reflector, which divided the reflector into two regions with different electric field strengths. The addition of the reflector not only increased the flight time of ions in limited space but also modified the initial divergence of ions, which significantly improved the resolution of TOFMS. Two microchannel plates (HAMAMATSY PHOTONICS F1552-011) were used in the MCP detector. The effective area of each MCP was 27 mm and the channel diameter was 12 μm. The bias angle was 12°. Voltage (800 V) was applied to each MCP via a resistive voltage divider. The detailed voltage parameters in CIFI−TOFMS are listed in Table 1. The output of the MCP detector of the TOFMS was passed to a time-digital converter (ORTEC 9353, 1 GHz burst rates, 100 ps time resolution). The extraction cycles of ions were 40 μs (25 kHz), and the corresponding recording range of mass-to-charge was 10–400 Th. The data processing software (National Copyright Administration of the People’s Republic of China, Computer Software Copyright Registration Certificate, Registration Number: 2022SR0835477; 2022SR0835476) was independently designed by our lab, which could process signal data from the time-digital converter in real-time and convert it into ion intensity. The software mainly included instrument control, mass calibration, spectrum processing, and long-term continuous data acquisition. In the current configuration, the size of the CIFI−TOFMS was 90 × 50 × 50 cm, excluding the dry scroll pump.

#### 2.2.4. Analytical Process

VOCs and OVOCs in the atmosphere entered the ionization source via a heated PEEK capillary under a pressure difference. During the sampling process, the temperature of the sampling capillary was maintained between 80 and 180 °C to avoid residual samples inside the pipeline. Ionization was achieved by direct photoionization or ion–molecule reaction reagent ions generated in the CIFI ionization source at a pressure of about 550 Pa. The mass calibration of the CIFI−TOFMS was achieved by sampling known substances (the 1 ppmv standard gas mixture of benzene, toluene, and p-toluene), measuring ion flight times, and converting them to mass-to-charge ratios. The mass calibration process was conducted every 2 weeks.

## 3. Results and Discussion

### 3.1. Enhanced Sensitivity and Soft Ionization for Samples with the CIFI Ionization Source

**Figure 3:**
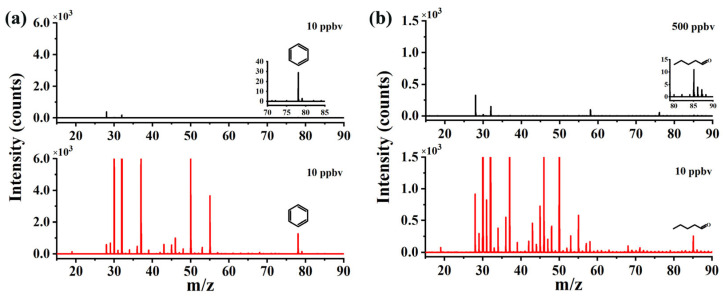
Enhancement effect of RF on (**a**) benzene and (**b**) pentanal (black, off; red, on).

An amount of 10 ppbv benzene and 500 ppbv pentanal was used to test the ionization ability of the CIFI ionization source with or without RF voltage, and the results are shown in Figure 3. Characteristic peaks of benzene and pentanal were molecular ion [M]^+^* of C_6_H_6_^+^* and [M − H]^+^ of C_5_H_9_O^+^, respectively. Ions are produced in three ways inside the CIFI ionization source: (1) Sample molecules are directly photoionized (Equation (1)). (2) Sample molecules can be ionized via ion–molecule reactions with O_2_^+^* reagent ions, namely chemical ionization (CI). The reagent ion O_2_^+^* used in CI is generated via the following pathways: a large number of high-energy photoelectrons are generated on the metal electrodes by the irradiation of VUV light, which collide with oxygen in the air under the acceleration of the electric field to produce O_2_^+^* reagent ions (Equations (2)–(4)). (3) Water in the air can also be ionized to generate (H_2_O)_n_·H_3_O^+^ reagent ions (n: 0–2). Therefore, a proton transfer reaction can occur between reagent ions and sample molecules (Equation (5)). In the equations, M represents sample molecules and hv represents ultraviolet photon.

(1) $$M+hv\to M^{+*}$$

(2) $$Metal+hv\to e^{-}$$

(3) $$O_{2}+e^{-}\to O_{2}^{+*}+2e^{-}$$

(4) $$O_{2}^{+*}+M\to M^{+*}+O_{2}$$

(5) $$H_{3}O^{+}\cdot {\left(H_{2}O\right)}_{n}+M\to M\cdot H^{+}+{\left(H_{2}O\right)}_{n+1}$$

So, in Figure 3, we can observe that the intensities of O_2_^+^* and [(H_2_O)_2_ + H]^+^ are about 11,000 counts s^−1^ and 2100 counts s^−1^, respectively. The CIFI ionization source is a chemical ionization device, and benzene molecular ions [M]^+^* of C_6_H_6_^+^* are produced from SPI and CI with O_2_^+^* reagent ions. Pentanal is firstly ionized to form odd-electron ions, and it can further undergo hydrogen rupture at the carbonyl α-position to generate [M − H]^+^ ions of C_5_H_9_O^+^.

When no RF voltage was applied to the segmented quadrupole, the signal intensity of 10 ppbv benzene was 29 counts s^−1^, and the signal intensity of 500 ppbv pentanal was 11 counts s^−1^. After turning on the RF voltage, that of 10 ppbv benzene was 1275 counts s^−1^, and that of 10 ppbv pentanal was 254 counts s^−1^. The signal intensities of benzene and pentanal increased by 44 and 1154 times, respectively.

The application of RF voltage on the segmented quadrupole is the main factor affecting the ion transport efficiency and sensitivity in this CIFI ionization source. The radial electric field of the segmented quadrupole causes ions to oscillate up and down, increasing the number of ion–molecule collisions, and cooling the ion beam onto the axis, thus improving the yield of sample ions and ion transport efficiency. When the RF voltage is turned off, the ionized ions are mainly transported by the DC electric field on the segmented quadrupole. At high pressure, the binding effect of the DC electric field on the ions is relatively weak, resulting in lower sensitivity of the instrument to detect benzene and pentanal. After applying the RF voltage, the ionized ions converge towards the axis under the constraint of the RF field, improving the ion transport efficiency.

**Figure 4:**
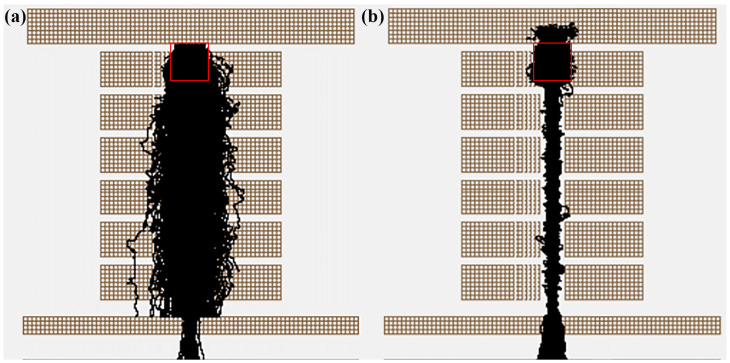
Ion trajectories of 100 Th ions with (**a**) RF on and (**b**) RF off (The red box represents the location of ions generation).

To understand the process of increased sensitivity in detail, we used SIMION 2020 to study how the RF electric field of the segmented quadrupole affects ion transport efficiency and chemical ionization efficiency initiated from ion–molecule collision frequency. The simulation of the effect of the electric field on ion trajectories in the CIFI ionization source is shown in Figure 4, and it can be intuitively seen that radio-frequency electric field can effectively improve ion transport efficiency. During the simulation process, the geometric resolution of the model was 0.5 mm gu^−1^, using a hard-sphere collision model [17]. An amount of 500 ions was randomly generated in a cylindrical area with a diameter of 4 mm and a height of 4 mm. The angle divergence of the ions was 10°, and the initial energy was 1 eV, as shown in Figure 4 (red box). The applied voltage conditions were as follows: V_2_ = 25 V, V_3_ = 20 V, V_4_ = 15 V, and S_1_ = 13 V.

**Figure 5:**
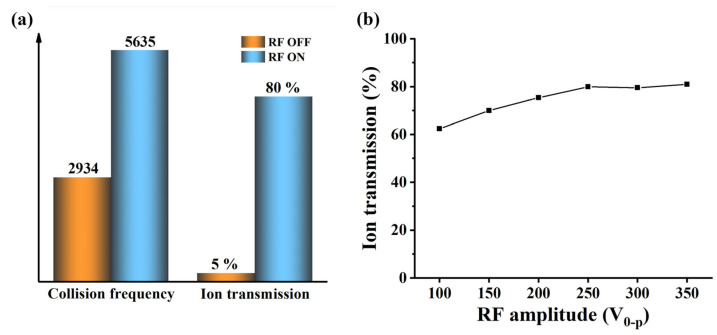
(**a**) The effect of applying or turning off RF on the ion transmission and the collision frequency between ions and neutral molecules; (**b**) simulation results of the influence of RF voltage on ion transmission.

The simulation results in Figure 5a indicate that the addition of RF voltage can not only significantly improve the ion transport efficiency, but also improve the collision probability between ions and background gas. Without the RF voltage, the number of collisions between ions and background gas is 2934; the transport efficiency of 500 ions is about 5%. After applying the RF voltage, the number of collisions between ions and background gas was 5635, an increase of 2701 compared to turning off the RF voltage; the transmission efficiency of 500 ions is 80%, which is a 16 times increase compared to the condition with RF voltage turned off. However, it can be observed that the improvement in ion transmission has a far greater impact than collision frequency. Hence, in order to further understand the impact of RF voltage on ion transport efficiency, we simulated ion transport efficiency under six different RF voltage conditions. The results in Figure 5b show that as RF voltage increases, the convergence effect of the ion beam towards the axis becomes more and more obvious. The ion transport efficiency increases with the increase in RF voltage and finally tends to flatten out.

**Figure 6:**
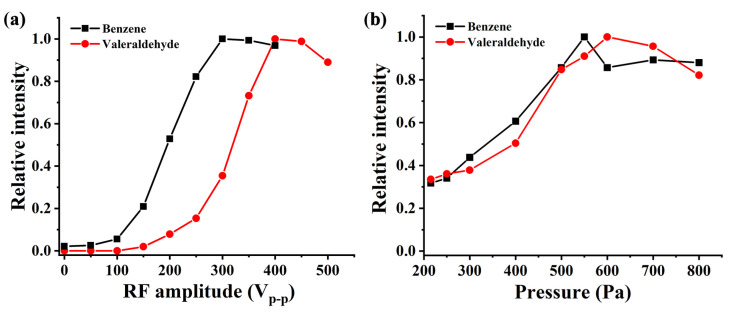
Sensitivity of (**a**) benzene and (**b**) pentanal under different RF voltage and pressure.

In order to further optimize the performance of the ionization source, experiments were conducted on the sensitivity of benzene and pentanal under different RF voltage values. The results are shown in Figure 6a. The RF voltage value has a significant impact on the sensitivity of benzene and pentanal. As the RF voltage value increases, the ion intensity gradually increases until the sensitivity stabilizes at V_p-p_ = 400 V, which is consistent with the simulated trend in Figure 5b. The mean free path of substances is closely related to pressure, so an optimization experiment was conducted to investigate the effect of ionization source pressure on ion intensity. The results shown in Figure 6b indicate that the optimal ionization source pressure for detection sensitivity of benzene and pentanal is around 550 Pa.

### 3.2. Linear Dynamic Range, Sensitivity, and Repeatability

**Figure 7:**
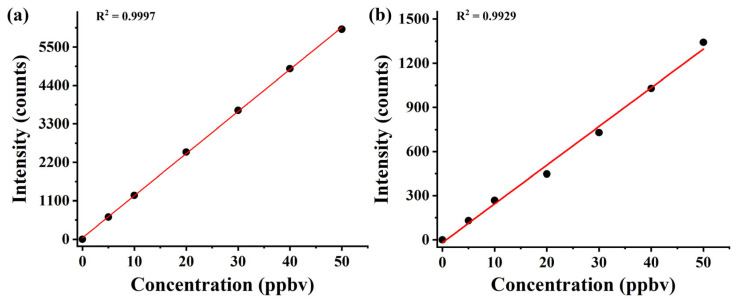
Calibration curves of (**a**) benzene and (**b**) pentanal with mixing ratios at the ppbv level.

In order to evaluate the performance of the CIFI ionization source, we investigated the response sensitivities and limit of detection (LOD) of the instrument to benzene and pentanal and evaluated its stability. The calibration curves of benzene and pentanal (Figure 7) were obtained by dynamically diluting the standard gas. The results for sensitivities and LODs are summarized in Table 2, with a detection sensitivity of 127.5 and 25.4 counts ppbv^−1^ for benzene and pentanal, and a LOD of 23.5 and 118.1 pptv, respectively.

**Figure 8:**
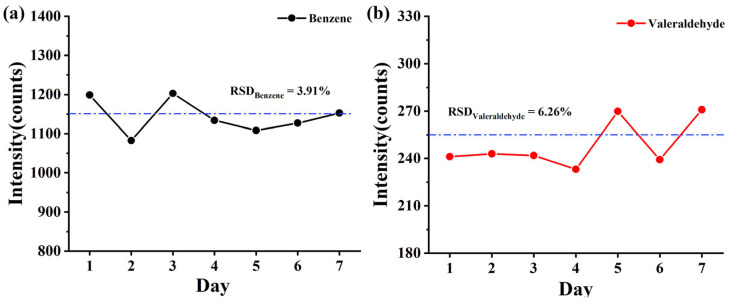
Average signal intensity of (**a**) benzene and (**b**) pentanal (10 ppbv) for 7 days (The dotted lines represent the average value of intensity).

The ionization source is crucial to the stability of the instrument. Therefore, stability experiments were conducted on TOFMS equipped with the CIFI ionization source. A 100 min continuous monitoring experiment was conducted daily by using 10 ppbv of benzene and glutaraldehyde for 7 consecutive days. Inter-day precisions were explored for benzene and glutaraldehyde with a relative standard deviation (RSD) of 3.91% and 6.26%, respectively, demonstrating satisfactory repeatability of the CIFI ionization source (Figure 8).

### 3.3. Application in Measurement of OVOCs in the Atmosphere

**Figure 9:**
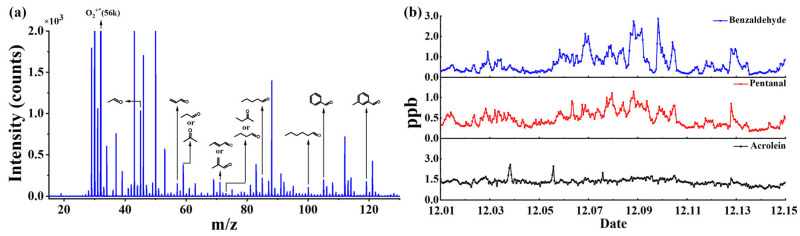
(**a**) Mass spectra of 2 ppbv OVOCs; (**b**) online monitoring data of benzaldehyde, pentanal, and acrolein in the atmosphere.

A mixture of 12 types of OVOC standard gases (acetaldehyde, acrolein, acetone, propionaldehyde, crotonaldehyde, methacrolein, 2-butanone, butanal, pentanal, hexanal, benzaldehyde, and m-tolualdehyde) was used to evaluate the detection performance of the CIFI ionization source for OVOCs. Figure 9a shows the mass spectra of OVOCs with an integration time of 5 s. In the CIFI ionization source, acetaldehyde, acrolein, acetone, propionaldehyde, crotonaldehyde, methacrolein, 2-butanone, and butanal mainly undergo proton transfer reactions to produce [M + H]^+^. The characteristic peak of hexanal was M^+^*, which was conducted via SPI and CI using O_2_^+^ reagent ions. Pentanal, benzaldehyde, and m-tolualdehyde were quite different; ionized odd-electron ions M^+^* could further undergo hydrogen rupture at the carbonyl α-position to generate [M − H]^+^. The sensitivity, LOD, and comparison results with other instruments are summarized in Table 3. The LOD of OVOCs using the TOFMS equipped with the CIFI ionization source can reach 6–200 pptv, demonstrating the potential of the CIFI ionization source for detecting OVOCs.

Based on the above experimental results, a 14-day online observation was conducted using the TOFMS equipped with the CIFI ionization source at Shandong University, Ji Mo District, Qingdao, Shandong Province, China, from 1 December 2022. During the monitoring period, the ambient air was pumped into the sampling inlet through the sampling pump, and partial air was introduced into the instrument through a bypass. Monitoring was conducted every hour (with an integration time of 1 min) for 14 consecutive days. The measurement results depicted in Figure 9b show the mixing ratios of benzaldehyde, pentanal, and acrolein in the air fluctuate within the range of 1–3 ppbv. This is consistent with the situation that there are no pollution emission sources such as chemical industrial parks near the school, so the air pollution is relatively small. The field observation results illustrate that CIFI−TOFMS is practical and useful for monitoring OVOCs in the atmosphere.

## 4. Conclusions

In this study, a CIFI ionization source was developed for the online measurement of OVOCs in the atmosphere, with a sensitivity in the pptv range. The CIFI ionization source not only improves ionization efficiency via more effective collisions of ion–molecules but also focuses ions to the central axis of the IMR and improves the ions’ transport efficiency. The structure and voltage application conditions of the CIFI were analyzed using SIMION 2020 simulation, and it was verified that the RF field in the ionization source focuses the ions radially to enhance the efficiency and sensitivity of ion transport. Finally, CIFI−TOFMS was applied to the online monitoring of OVOCs in the atmosphere, and the mixing ratios of benzaldehyde, pentanal, and acrolein were presented in the ppbv range, which proves the potential of the CIFI ionization source in the online monitoring of OVOCs in the atmosphere.

## Figures and Tables

**Table 1:** System parameters of CIFI−TOFMS.

Ion Source	Parameters
vacuum ultraviolet lamp	1100 V; 0.55 mA
repulsion electrode (V_1_)	30 V
extraction electrode (V_2_)	25 V
voltage at the front end of the segmented quadrupole (V_3_)	20 V
the segmented quadrupole RF frequency and magnitude	2.1 MHz; 300 V_P-P_
voltage at the back end of the segmented quadrupole (V_4_)	15 V
Ion Transmission System	Parameters
the first differential vacuum orifice (S_1_)	13 V
RF-only quadrupole RF frequency, magnitude, and DC float	1.8 MHz; 280 V_P-P_; 8 V
the second differential vacuum orifice	5 V
the ion lens	−20 V; −50 V
TOF Mass Analyzer	Parameters
slit	2 × 8 mm
modulator	25 kHz frequency, ±480 V pulse voltage
accelerator	62.8 mm in length; −3800 V
drift region	468 mm in length
reflector	reflector 1, −200 V; reflector 2, 1020 V
microchannel plate detector	−4000 V

**Table 2:** Detection sensitivity and LODs.

Compound (*m/z*)	Sensitivity (cps ppbv^−1^)	LOD (pptv)
Benzene (78)	127.5	23.5
Pentanal (85)	25.4	118.1

**Table 3:** Detection sensitivity, LOD, and substances information.

Compounds	Molecular Formula	Characteristic Ions	Linear Range (ppbv)	LOD (pptv)	PTRMS [18] LOD/0.1 s (pptv)	SPIMS [18] LOD/5 s (ppbv)	IE (eV)
Acetaldehyde	C_2_H_4_O	*m/z* = 45; [M + H]^+^	2–40	6	138	--	10.22 [19]
Acrolein	C_3_H_4_O	*m/z* = 57; [M + H]^+^	2–40	45	27	22.5	10.10 [20]
Acetone	C_3_H_6_O	*m/z* = 59; [M + H]^+^	2–40	17	22	3.62	9.71 [21]
Propionaldehyde	C_3_H_6_O				--	--	9.82 [22]
Crotonaldehyde	C_4_H_6_O	*m/z* = 71; [M + H]^+^	2–40	36	12	2.6	9.73 [23]
Methacrolein	C_4_H_6_O				--	--	9.92 [24]
2-Butanone	C_4_H_8_O	*m/z* = 73; [M + H]^+^	2–40	200	--	--	9.70 [25]
Butanal	C_4_H_8_O				--	--	9.83 [26]
Pentanal	C_5_H_10_O	*m/z* = 85; [M−H]^+^	2–40	44	56	22.6	9.74 [27]
Hexanal	C_6_H_12_O	*m/z* = 100; M^+^*	2–40	57	34	22	9.64 [28]
Benzaldehyde	C_7_H_6_O	*m/z* = 105; [M−H]^+^	2–40	31	--	--	9.49 [29]
m-Tolualdehyde	C_8_H_8_O	*m/z* = 119; [M−H]^+^	2–40	42	--	--	--

Limits of detection (LOD) are calculated for 5 s integration time.

## Data Availability

Not applicable.
